# Weight Factor as a Parameter for Optimal Part Orientation in the L-PBF Printing Process Using Numerical Simulation

**DOI:** 10.3390/ma17143604

**Published:** 2024-07-22

**Authors:** Ľuboš Kaščák, Ján Varga, Jana Bidulská, Róbert Bidulský, Diego Manfredi

**Affiliations:** 1 Department of Technology, Materials and Computer Supported Production, Faculty of Mechanical Engineering, Technical University of Košice, Letná 9, 04002 Košice, Slovakia; jan.varga@tuke.sk; 2 Department of Plastic Deformation and Simulation Processes, Institute of Materials and Quality Engineering, Faculty of Materials, Metallurgy and Recycling, Technical University of Kosice, Vysokoskolska 4, 04200 Košice, Slovakia; jana.bidulska@tuke.sk; 3 Bodva Industry and Innovation Cluster, Budulov 174, 04501 Moldava and Bodvou, Slovakia; director@biic.sk; 4 Advanced Research and Innovation Hub, Budulov 174, 04501 Moldava and Bodvou, Slovakia; 5 Department of Applied Science and Technology, Politecnico di Torino, Corso Duca degli Abruzzi 24, 10129 Torino, Italy; diego.manfredi@polito.it

**Keywords:** metal additive process, simulation process, support material, voxel elements

## Abstract

The L-PBF process belongs to the most modern methods of manufacturing complex-shaped parts. It is used especially in the automotive, aviation industries, and in the consumer products industry as well. Numerical simulation in the powder sintering process is a means of optimizing time efficiency, accuracy and predicting future errors. It is one of the means to optimize the L-PBF process, which makes it possible to investigate the influence of individual parameters on additive manufacturing. This research makes it possible to predict the correct orientation of a part based on selected criteria, which are assigned a weighting factor in the form of parameters with which the simulation software Simufact Additive can work. Based on these, three possible orientations of the part were analysed with respect to the area of the supporting material, the volume of the supporting material, the number of voxels, and the building risk. Finally, the results of a simulation and the results of the tensile test were compared. From the results of the static tensile test, as well as from the results of the numerical simulation, it was found that better characteristics were achieved for the orientation of part no. 1 compared to orientation of part No. 3.

## 1. Introduction

The production of various parts in the metal additive manufacturing process requires knowledge not only of the powder sintering process itself and the influence of parameters on the quality of the future part, but also of the importance of material consumption, which is closely related to the orientation of the part during the build process [1]. The correct orientation of the part plays an important role in the construction of the model, mainly from the point of view of the time interface, which defines the length of time spent on the production of the part.

The importance of predicting the appropriate orientation of the part for the metal additive manufacturing process is in achieving the potentially lowest deformations and residual stresses. The orientation of the part should be chosen so that the cross-sectional area remains as even as possible along the direction of growth (z). The orientation of the part also affects the surface roughness [2].

Ma et al. [3] attempted to quantify residual stresses by designing geometries that deform predictably, including the bridge curvature method (BCM), where they used AlSi10Mg as the material. The printing process was evaluated by the hole drilling method and simulated using ANSYS and Simufact Additive software. Similar research was conducted by Thakur et al. [4], who analyzed the optimal values of laser power, scanning speed and cavity spacing to reduce the residual stress and distortion in the SLM (selective laser melting) process to an acceptable extent. Simufact Additive and ANSYS Additive were used as simulation tools. As a result, optimal process parameters, such as laser power 80 (W), scanning speed 950 (mm/s) and hatch spacing 70 (μm), were obtained.

One way of achieving a high-quality part with the required dimensional and functional properties is by implementation of several trials/iterations to find out which input parameters are suitable for the production process. However, due to several factors that affect the metal additive manufacturing process (e.g., powder thickness, scanning speed, chamber temperature, machine performance), as well as other parameters, such as the orientation of the part structure, the amount of support material that fulfills the function of cooling, etc., this can be a time-consuming task. The criteria that determine the rapid generation of many feasible alternatives for the optimal construction of the part with respect to the support area, support volume or so-called build risk are very important for the process. They allow us to identify the risk of building layers based on geometry and orientation. The greater the change in area from layer to layer, the higher the risk of problems during the building process.

Simulation tools are increasingly being used in various technologies, which make it possible to predict various errors, not only in the production process but also in the process of joining different materials [5,6], and the influence of each parameter that enters the process [7]. Their use makes it possible to analyze the production process in a short period, which would otherwise require a long time by retesting [8,9,10,11]. In their research, Kaščák et al. [12] used the Simufact Additive software, where the goal was to predict the behavior of the material in the metal additive manufacturing process. In their study, they focused on the differences in the form of the achieved volume share within the support generation both with and without the optimization function. Kamarudin et al. [13] researched the influence of the orientation of the part at different angles on the build platform during the building process. In their work, they evaluated the geometric texture of the surface and used AlSi10Mg as a powder.

Likewise, for the prediction of possible errors in the production of test samples, Kaščák et al. [14] used a simulation program which dealt with the simulation of 316L stainless steel intended for the LPBF process. The result of the application of the numerical simulation was the prediction of possible deformations and errors of the part by analyzing the resulting distortion when reaching the maximum deviation values of −0.01 mm and −0.13 mm. The effect of the orientation of the part on its building and surface quality, microstructure and mechanical properties with selective laser melting of stainless steel 316L was investigated in the work by Alsalla et al. [15]. The results indicated that the part building orientation influences the microstructure of the parts, which, in turn, affects their mechanical properties and surface quality. The orientation of the part, as well as the density or the pattern of the filling, must also be considered because they contribute to the achievement of a high-quality part in the production process [16].

The orientation of the part in the building process also contributes to the maximization of self-supporting surfaces, which was addressed in the study by Leutenecker et al. [17]. Morgan et al. [18] also addressed this important factor and the influence of orientation. The effectiveness of the orientation of the part structure in the production of 3D-printing intended for material extrusions, and also powder bed fusion, was investigated by Salmi et al. [19], who emphasized that the production time per part largely depends on the geometry, orientation, printing process and the number of parts produced in one assembly.

Distortion prediction and compensation in the selective laser melting process were addressed in the work by Afazov et al. [20], who applied a simulation program, with the results showing that a reduction in deformation in this process is now possible with industrial macro-components. Effective simulation of the additive manufacturing process was investigated by Song et al. [21], who tried to predict the residual stresses and distortions in complex metal components through simulation. The results showed that simulation of the surrounding powder bed was important for accurately predicting the temperature history for complex support material geometries. Mayer et al. [22] applied AN-SYS Additive Print and ANSYS Additive Suite simulations to validate residual deformations in the additive manufacturing of metal parts. Validation of the programs was carried out based on different geometries of samples with different wall thicknesses and deformation characteristics.

The correct selection of a simulation program whose calculation algorithms can process all the information and input data available to the L-PBF process is very difficult. It is important to know the possibilities of this software so that we can process, evaluate, and thus offer the best solutions to build a part in metal additive manufacturing as easily and efficiently as possible. These simulation tools should be able to predict errors that may occur in the building process [23,24,25,26]. Even though many simulation tools are still being developed, it is very important to choose one that can quickly and efficiently process the input data affecting the L-PBF process. Based on the input data, it is thus possible to predict results such as stresses and deformations in the part [27,28,29].

As a result, the entire simulation process can be accelerated within the framework of obtaining the necessary data for future production of the part, which results in a high-quality part with the required dimensions [30,31,32,33,34]. In Majeed et al. [35], simulation was used to analyze the behavior of layers in the process of building a metal part in connection with the definition of various input parameters. Budinoff et al. [36] applied a simulation approach as a tool to investigate how geometry and topology affect additive manufacturing process costs and associated post-processing operations. A cost model implemented in the simulation process identified differences of up to 14% between the cheapest and most expensive design alternatives. Parameters such as the weight of parts and building time were the most influential factors in the various designs.

The main goal of this research was to predict the optimal orientation of the part with the help of the Simufact Additive simulation software, taking into account the selected criteria necessary for the construction process (such as support area, support volume and building risk) and with input parameters such as laser power, scanning speed, layer thickness, hatch distance and building platform temperature. Applying a suitable simulation tool for error prediction, even before the production process, can help designers or process engineers consider the degree of efficiency by assigning a specific weighting factor. They can achieve a more efficient process without increasing the error rate on the manufactured part.

This study aimed to investigate the suitability of using simulation software concerning the definition of weighting factors intended for three different orientations of the part. By using the definition of the weighting factor, it is possible not only to effectively avoid excess time in the production of the optimal orientation of the part but also to find out which orientation of the part would create less material waste. The amount and volume of the carrier material have a significant impact on the printing process and the result. This contributes to the expansion of knowledge about the effects of von Mises stresses on the orientation of the part.

There appears to be a lack of a simulation tool in the area of metal additive manufacturing that would address the complexity of data processing sufficiently such that it could use its functions to achieve the necessary information more effectively for the production process. Effective simulation does not consist of repetitive calculations, but using the right functions should avoid repetitive simulations considering what we need to achieve. These may include using a smaller volume of support material, consideration of the quality of the part, the risk of construction, the orientation of the part, etc. This can be helped by defining a weighting factor that allows for assessing the degree of importance that the simulation tool should focus on.

## 2. Materials and Methods

The following steps comprise the methodology:

- Prediction of the production orientation of the part based on selected criteria, which were assigned a weighting factor.
- Analysis of the orientation of the part and its influence on the critical angle for forming the part support, support generation view, volume fraction and von Mises stresses.
- Verification of the numerical simulation results.

### 2.1. Numerical Simulation

**Figure 1:**
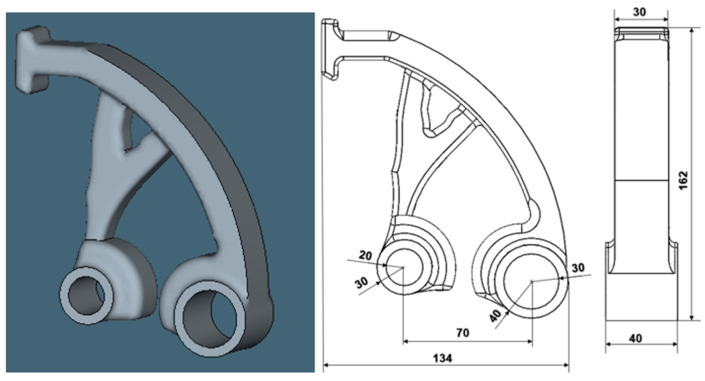
Selected part of the simulation process.

Figure 1 shows the part examined to compare the simulation results concerning the orientation of the part (134 × 162 × 40 mm), which was assigned a specific material in the form of AlSi10Mg powder. The simulation was carried out for the metal additive manufacturing process known as laser powder bed fusion on an EOSINT M270 Dual Mode Machine (manufactured by EOS, Krailling, Germany), and a cylindrical geometry was used to create the support structure. The numerical simulation was carried out using the software Simufact Additive 2022 (Hexagon Manufacturing Intelligence GmbH, München, Germany).

For the L-PBF process, a mechanical configuration working on the principle of inherent strain was chosen. The simulation itself was assigned a simulation type of “manufacturing”, which consisted of three levels, with each level defining a specific phase of the process. In this research, phase 1 is Build, phase 2 is Cutting, and phase 3 is Support removal. For simulation purposes, the Simufact Additive 2022 program used voxel elements that formed hexahedral elements that represented the part itself together with a combination of the volume fraction of the part. The Simufact Additive 2022 program made it possible to use different criteria to obtain the most suitable orientation when the parts are rotated around the global X- and Y-axes during the calculation, and criteria are calculated for each orientation. The calculation was not performed based on simulations but based on geometric criteria. The orientation assistant thus made it possible to select two areas, namely, the property area considering the resolution, and the area considering the distance of the geometry of the part from the build platform. In the case of resolution, it was possible to choose four options: minimum, coarse, medium, and smooth, while a finer resolution leads to a larger number of data points and, therefore, to a longer calculation time. In our case, a medium resolution was chosen.

In the case of defining specific criteria, three default criteria were available that could be used to determine the best possible orientation for the building part:

- A support area that calculates the sum of all support surfaces.
- Support volume, which calculates the sum of all the support volumes.
- Building risk, which calculates the level of building risk based on the current orientation of the part.

For each type of criterion, it was possible to define a parameter called the weighting factor, which could be chosen in the range from 1 to 10. In our case, the value 1 was chosen, which means that all the criteria had the same weight. The above criteria are used to calculate one global value result concerning the defined weighting factors. This result was visualized using a standard color legend. The best orientation is shown in green. The worst orientation is shown in dark red.

For the simulation, the same input parameters based on the values of inherent strain εxx = −0.0058, εyy = −0.0022, εzz = −0.03 were chosen. The process parameters for the simulation are listed in Table 1.

**Figure 2:**
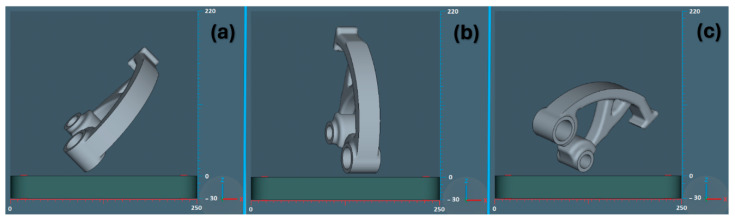
Part orientation plane XZ used in simulation: (**a**) orientation 1, (**b**) orientation 2, (**c**) orientation 3.

In the simulation, a phase was implemented simulating a cut through the supports in one direction for all three orientations to disconnect the part/supports from the base plate. The distance of the model from the background for band sawing in the *Z*-axis was defined as 1 mm. Three different orientations of the part according to the dimensions of the EOSINT M270 machine (Figure 2) were compared with each other concerning the three selected criteria for each orientation of the construction of the part, namely, the area of support, the volume of support, and the build risk. At the same time, the distribution of the support structure and the resulting properties in the form of the number of voxel elements, the number of nodes, and the number of individual layers were also compared.

### 2.2. Tensile Test

Due to the high thermal gradients occurring during the sintering process, the individual parts were exposed to the annealing process at a temperature of 300 °C for 2 h using a machine Ecoblast/F, produced by Silco S.r.l., Rivalta di Torino, Italy. After that, the samples were taken for implementation of the tensile test on an EASYDUR 3MZ-5000 machine (Producer Easydur, testing equipment, Arcisate, Italy), where the speed of the transverse head was 2 mm/min and the deformation was measured with a piezoelectric extensometer. For the tensile test, 3 samples were used according to the ASTM E8M standard [37] for orientations 1 and 3, which proved to be the best and worst variants within the simulation.

## 3. Results

### 3.1. Analysis of Numerical Simulation Results

In the first step, the part was imported into the environment of the simulation program Simufact Additive 2022 for comparison purposes concerning the orientation of the part. Each proposed orientation of the part resulted in an efficient or less efficient build process, either within the support or of the critical angle during building, considering the criteria that were determined for the build process.

**Figure 3:**
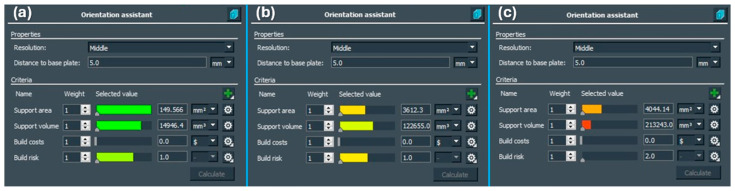
The optimal criteria for the build process: (**a**) orientation 1, (**b**) orientation 2, (**c**) orientation 3.

From the point of view of the individual criteria chosen for the build purpose, including the support areas, support volume and build risk, the best possible variant for the additive process was calculated meeting the above criteria. The criteria for the building process concerning the orientation of the part are shown in Figure 3.

**Figure 4:**
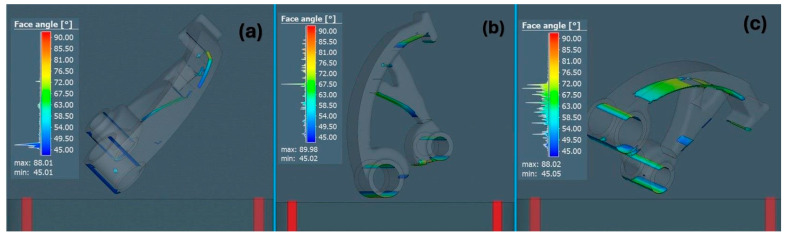
The critical angle for forming part support: (**a**) orientation 1, (**b**) orientation 2, (**c**) orientation 3.

For a better graphical interpretation of the areas representing the need for a support structure to build the part, all three orientations were compared with each other, as shown in Figure 4, while a color scale defining a specific angle on the surface of the part is also shown. An angle of 45° was chosen as a parameter defining the critical angle of the surface.

**Figure 5:**
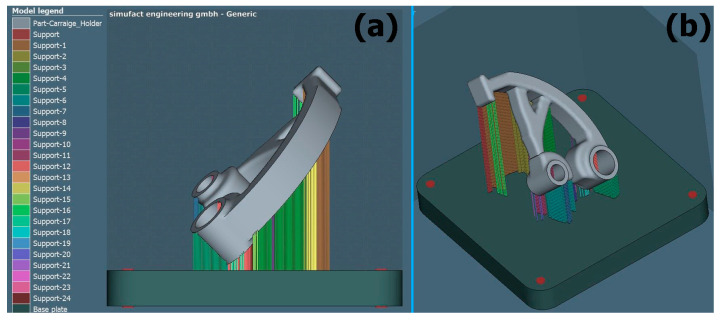
Support generation view for the orientation of part 1: (**a**) XZ plane, (**b**) 3D view.

**Figure 6:**
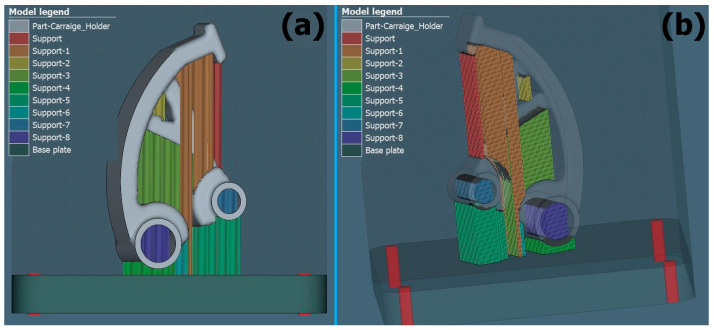
Support generation view for the orientation of part 2: (**a**) XZ plane, (**b**) 3D view.

**Figure 7:**
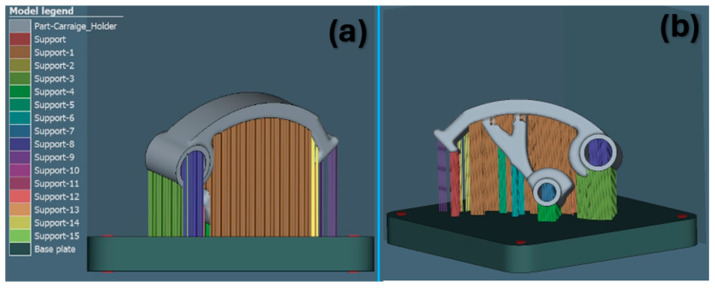
Support generation view for the orientation of part 3: (**a**) XZ plane, (**b**) 3D view.

Figure 5, Figure 6 and Figure 7 show support renderings for a specific orientation of the part in the case of its construction for the metal additive manufacturing process. In individual comparisons, it is possible to see the difference in the rendering of the support structure, as well as in the density of the created support.

From the individual comparisons, it was possible to see the differences in the distribution of the creation of the support structure concerning the orientation of the building part. The least supporting material was achieved with orientation 1, which for all three criteria predicted the optimal outcome, i.e., green color, as shown in Figure 3.

**Figure 8:**
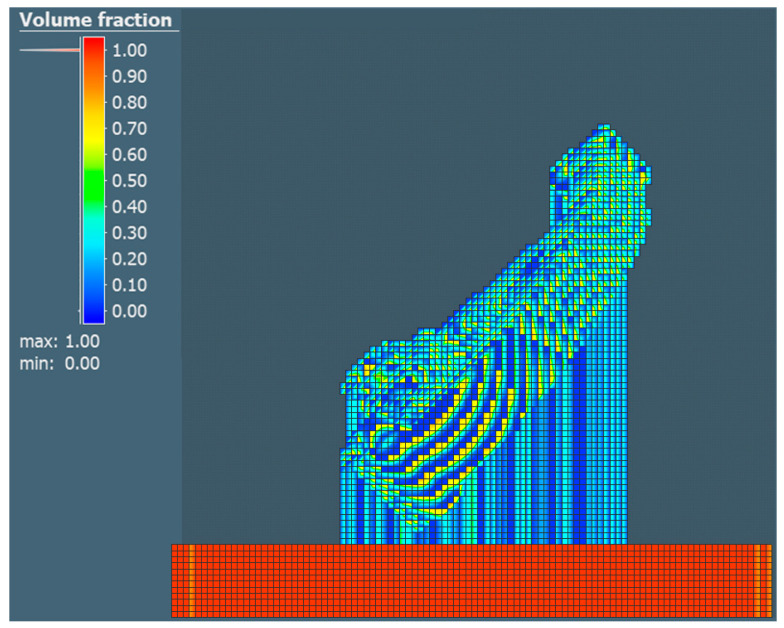
Volume fraction for the orientation of part No. 1.

**Figure 9:**
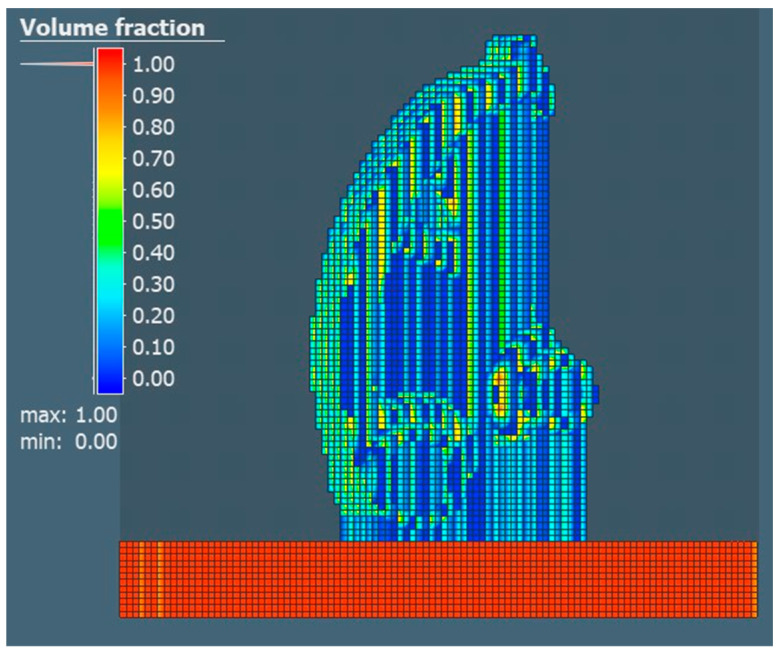
Volume fraction for the orientation of part No. 2.

**Figure 10:**
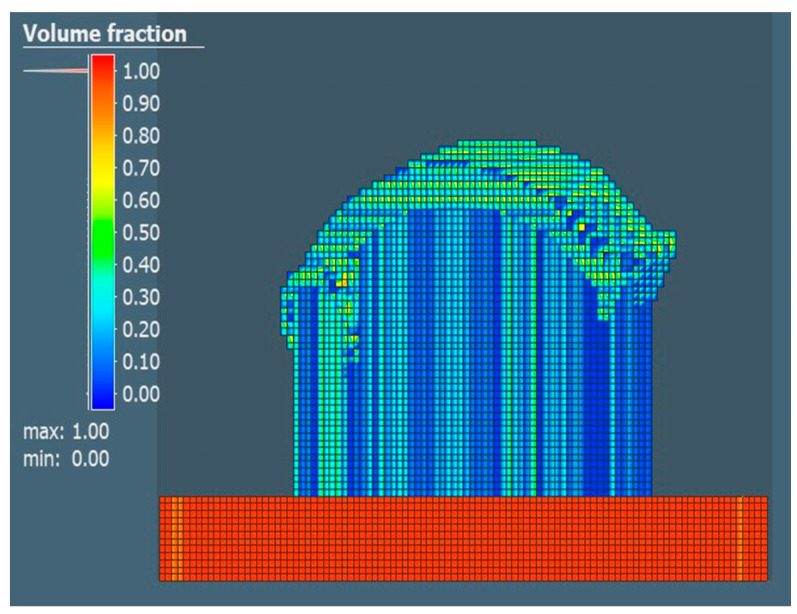
Volume fraction for the orientation of part No. 3.

Subsequently, the property defining the volume fraction of the part was realized and compared with other properties, the value of which defines how much of the voxel element volume is filled with geometry. A value of 2.42 mm was set for the surface mesh and a value of 2 mm was set to define the voxel element size for the X, Y and Z directions. The choice of the given value considered the short calculation time. Individual comparisons of the volume share for each of the selected orientations are shown in Figure 8, Figure 9 and Figure 10.

**Figure 11:**
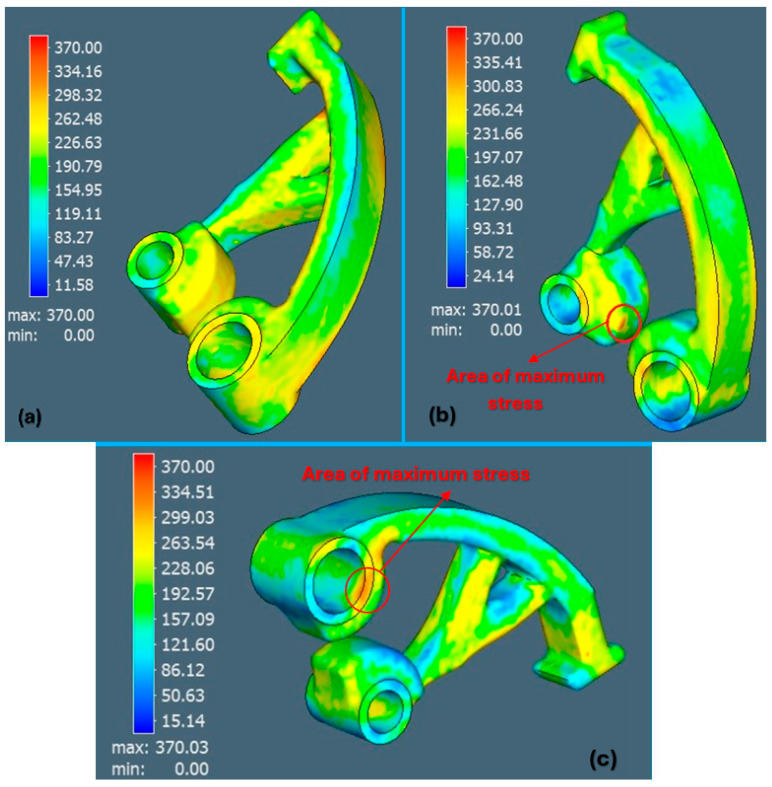
View of von Mises stresses: (**a**) orientation 1, (**b**) orientation 2, (**c**) orientation 3.

The last analysis within the simulation was the von Mises stresses (Figure 11), which showed stress values between 298 and 334 MPa (which represent the maximum values for all three orientations, approaching the yield limit of the material 370 MPa). The orientation of part No. 1 showed better results.

### 3.2. Results from Tensile Test

To verify the results obtained from the simulation, a static tensile test was carried out. The average values obtained of the mechanical properties of the samples concerning orientations 1 and 3 are shown in Table 2.

## 4. Discussion

The simulation program Simufact Additive 2022 predicted the optimal orientation of the part with respect to the selected criteria for the building part process, such as the area of support, the volume of support, and the build risk.

Mikulikova et al. [38] applied up to eight possible orientations of the part to obtain a suitable place for printing on the platform of the building board, whereby she compared the parameters considering the area content of the support material, the volume of the supporting material and the build risk. The use of Simufact Additive 2022 simulation software appears to be a more effective tool in this context. By using it, it was possible to find a suitable orientation of the part, which met all the conditions above defined by Mikulikova et al., thanks to the pre-selected criteria and the assignment of the weighting factor. Therefore, it was not necessary to calculate so many iterations before arriving at the result.

With regard to the appropriateness of using the Simufact Additive 2022 simulation program, based on our results presented in Table 3, it is possible to confirm the simplicity and effectiveness of obtaining the necessary results considering the influence of the orientation of the part on the selected criteria.

Similarly, Pagač et al. [39] had to use up to nine different position orientations in simulation software to find the most suitable orientation of the part to predict the distortion of the part in the L-PBF process for stainless steel 316L, with the same parameters that Mikulikova et al. investigated [38]. In this case, too, it can be noted that by applying specific criteria for the L-PBF process in the Simufact Additive 2022 simulation program, it is possible to achieve a more efficient evaluation of the necessary results and thus reduce the calculation time, which ultimately allows us to work with data/results faster and more efficiently to optimize the production process.

As noted by Gao et al. [40], the amount of material consumed, as well as the support area and support volume obtained concerning the orientation of the part in the process of metal additive manufacturing, affect the amount of material used, and, therefore, it is advisable to eliminate inappropriate orientations of the part due to various attempts or mistakes.

Bassoli et al. [2] applied the Simufact Additive simulation software for the part-building process to find the orientation of the part in the design chamber that would correspond to better dimensional accuracy. They realized several orientations of the part to predict the most suitable structure, considering not only the volume of the supporting material but also the von Mises stresses. Applying the weighting factors that we present in our study leads to more efficient identification of the appropriate orientation of the part.

Based on the obtained results from the tensile test (Table 2) and the results from the numerical simulation (Table 3), it can be concluded that the correct orientation of the part affects the mechanical properties of the material. The results imply that it is essential to obtain sufficient information about whether the part meets or does not meet possible criteria in connection with its functionality or the stress to which the future part will be exposed. The static tensile test showed better values for the orientation of part no. 1 compared to orientation No. 3, which corresponded to the simulation results.

## 5. Conclusions

The subject of the research was the use of simulation software in the process of metal additive manufacturing and the use of selected criteria for building the model. Based on the results, the simulation program Simufact Additive 2022 identified the most suitable orientation of the part during its additive manufacturing. It can be noted that even a simple factor, such as the orientation of the part, is of importance in the metal additive manufacturing process. Further research in this area will be aimed at supplementing the results from assessment of deviations in the form of shape and dimensional accuracy through digitization.

Consideration of the individual criteria that should be considered when designing the orientation of the part enabled achievement of the optimal orientation of the part in the build process. The comparison of the selected orientations shows that the most suitable parameters were achieved for orientation 1 in terms of the number of voxel elements, the number of nodes and the values defining the criterion of the area of the support, the volume of the support and the risk of creating parts.

The orientation number 3 showed the highest number of values defining the sum of all support surfaces, the sum of all support volumes, the number of voxel elements, or the number of nodes during the building of the part. In orientation 3, the construction risk value was 2. The construction risk predicts the print quality and thus points to problematic printing of the part in terms of possible occurrences of defects on the surface, occurrence of deformation, etc.

## Figures and Tables

**Table 1:** Process parameters intended for production simulation.

Power [W]	Scanning Speed [mm/s]	Layer Thickness [µm]	Hatching Distance [mm]	Building Platform Temperature [°C]
200	800	30	0.08	80

**Table 2:** Mechanical properties of the samples with orientations 1 and 3.

Orientation	Yield Strength σ0.2 [MPa]	Ultimate Tensile Strength σUTS [MPa]	Elongation at Break [%]
1	241 ± 4	326 ± 2	5.8 ± 0.3
3	219 ± 3	309 ± 3	4.3 ± 0.2

**Table 3:** Results considering the influence of the orientation of the part concerning the selected criteria.

Orientation	Support Area [mm^2^]	Support Volume [mm^3^]	Build Risk	Voxel Number	Nodes Number	Layer Number
1	149.566	14,946.4	1	41,909	66,449	70
2	3612.3	122,655.0	1	44,086	64,283	81
3	4044.14	213,243.0	2	58,397	84,159	51

## Data Availability

The raw data supporting the conclusions of this article will be made available by the authors on request.
